# 

**Published:** 1972-07

**Authors:** 


					,)!
tf<3<23
/?'IP *7
'c ' <973 j:
??UAig yy
BRISTOL
MEDICO- ,
CHIRURGICAL
JOURNAL
JULY 1972
VOL. 87 (Ih) No. 323

				

